# Scapular Dyskinesis and Associated Factors in Adult Elite Swimmers

**DOI:** 10.3390/medicina61101885

**Published:** 2025-10-21

**Authors:** Se Young Joo, Young Kyun Kim

**Affiliations:** Graduate School of Sports Medicine, CHA University, Seongnam 13496, Republic of Korea; rissa234@naver.com

**Keywords:** scapular dyskinesis, swimming, shoulder joint, shoulder pain

## Abstract

*Background and Objectives*: Swimmers are repeatedly exposed to overhead shoulder movements, which overload the surrounding soft tissue and may contribute to shoulder pain. These repetitive demands have also been implicated in the development of scapular dyskinesis (SD). This cross-sectional study aimed to determine the prevalence of SD and to examine its associations with extrinsic and intrinsic factors in adult elite swimmers. *Materials and Methods*: Fifty competitive swimmers (mean age, 23.9 years; mean training experience, 13.6 years) participated in this study. SD was graded using the Scapular Dyskinesis Test. Extrinsic factors included dominant side, breathing side, years of experience, and primary stroke. Intrinsic factors included Lateral Scapular Slide Test (LSST) distance, pectoralis minor length, glenohumeral internal rotation (IR) range of motion (ROM), shoulder pain, and Penn Shoulder Score. *Results*: SD was identified in 46% of swimmers. Years of experience and primary stroke showed no significant association with SD; however, obvious SD was observed only in butterfly and freestyle specialists. Increasing SD severity was associated with shorter pectoralis minor length (*p* < 0.001) and reduced IR ROM (*p* = 0.013), particularly in the obvious group. Although SD was not related to shoulder pain, it was significantly related to lower Penn Shoulder Scores (*p* = 0.039). *Conclusions*: SD is common in adult elite swimmers and is associated with shortened pectoralis minor, reduced IR ROM, and impaired shoulder function, but not to pain.

## 1. Introduction

Swimming requires repetitive overhead movements of the upper extremities [1] that places high functional demands on the shoulder joint [2]. Elite swimmers cover ~14,000 m per day, and perform about 2500 shoulder rotations per training session and ~16,000 rotations per week [3]. Such excessive shoulder rotations overload the surrounding soft tissues of the shoulder, and cause pain not only during swimming but also in daily activities; indeed, shoulder pain is the most common orthopedic injury among swimmers [4]. Repetitive overhead activities in swimming induce fatigue in the rotator cuff, scapular stabilizers, and anterior and posterior shoulder muscles [5], which can confer scapular instability and reduce the scapulohumeral rhythm [6]. These alterations in scapular kinematics are considered clinically important factors in swimmers, as they not only reduce the efficiency of shoulder function but are also associated with shoulder pathology [7].

Abnormalities in the static position or dynamic movement of the scapula constitute scapular dyskinesis (SD) [8]. SD has been associated with glenohumeral internal rotation deficit (GIRD) and SICK scapula syndrome [8,9]. Moreover, altered scapular positioning and/or movement place undue stress on the anterior shoulder structures and increases the posterior ‘peel-back’ of the biceps on the glenoid labrum [10]. Such altered mechanics increase stress on the biceps-labral complex and may contribute to secondary lesions involving the biceps pulley system. Biceps pulley lesions are often accompanied by partial articular supraspinatus tendon avulsion (PASTA) lesions [11]. Collectively, these mechanisms underscore that SD represents a clinically important condition in swimmers. The prevalence of SD is 54.5% and 33.3% in athletes participating in overhead and non-overhead sports, respectively [12]. In swimmers, SD prevalence ranges from 8.5% to 69% [5,13,14]. Previous studies included young swimmers whose osseous and soft-tissue adaptations to repetitive swimming movements were not yet fully developed [15]. Notably, SD was observed in 82% of swimmers who initially presented without SD after completing a 100 min swimming session [16]. Therefore, assessments of SD in adult elite swimmers should be conducted with sufficient training history, under resting conditions that exclude the influence of transient fatigue [13].

In swimmers aged 13–25 years, shoulder impingement syndrome and supraspinatus tendinopathy were reported in 90% and 69%, respectively [1]. Furthermore, shoulder joint laxity was observed in all 42 Olympic swimmers [17]. SD is associated with shoulder pathologies, such as shoulder impingement syndrome, rotator cuff disorders, and shoulder instability [7]; thus, SD may occur concurrently with shoulder injuries in swimmers. SD has been identified in 8.5% of 694 asymptomatic swimmers [13], which indicates the possibility that non-pathological factors, such as anatomical structures or functional characteristics, may contribute to its occurrence. However, few studies have analyzed the relationship between these factors and SD in swimmer populations.

Although previous studies have reported the prevalence of SD in swimmers [5,13,14], few have comprehensively analyzed its prevalence and associated factors in adult populations. A previous study examined the associations between SD and several extrinsic factors (e.g., dominant side, breathing side, primary stroke, and years of experience) in swimmers [10]; however, their participants were adolescents, and intrinsic factors were not included. Therefore, this study aimed to investigate the prevalence of SD in adult swimmers and to analyze its associations with extrinsic (dominant side, breathing side, years of experience, and primary stroke) and intrinsic (lateral scapular slide test [LSST] distance, pectoralis minor length, internal rotation [IR] range of motion [ROM], shoulder pain, and subjective shoulder function level) factors. The hypotheses of this study were that: (1) the occurrence patterns of SD would differ according to breathing side and primary stroke; (2) a longer athletic career would be associated with worse SD grade; and (3) worse SD grade would be accompanied by increased LSST distance, shortened pectoralis minor length, decreased IR-ROM, increased shoulder pain, and reduced subjective shoulder function.

## 2. Materials and Methods

### 2.1. Participants

This single-blind cross-sectional study enrolled 50 swimmers from five teams, all of whom were either national team or competitors in the national sports competition. All participants engaged in year-round, high-volume, and structured training programs characteristic of elite swimmers. The inclusion criterion was adult swimmers registered in 2024 with the Korea Swimming Federation. None had a history of recent shoulder surgery or any acute injury that could have interfered with swimming or testing performance. The sample size was calculated using G*Power software version 3.1 (University of Kiel, Kiel, Germany), based on an effect size of 0.54 reported in a previous study [18], a significance level of 0.05, and a statistical power of 0.80, necessitating a minimum sample size of 39. All participants provided written informed consent. The study was approved by the Institutional Review Board (IRB) of CHA University (approval number: 1044308-202412-HR-223-02) and conducted in accordance with the ethical principles of the Declaration of Helsinki.

### 2.2. Procedures

Participant characteristics (e.g., sex, age, height, weight, and dominant side) and swimming-related information (e.g., years of experience, breathing side, primary stroke, and race distance) were recorded. The dominant side was defined based on the hand or arm that was primarily used in daily activities. Shoulder pain, satisfaction, and the functional level were assessed using the Penn Shoulder Score questionnaire, followed by the scapular dyskinesis test (SDT) to evaluate SD. Next, the LSST was conducted to measure the distance between the thoracic spine and the scapula. Internal and external rotation ROM of the shoulder joint were measured with the shoulder abducted to 90° and the elbow flexed to 90°, corresponding to the ER2 testing position, and pectoralis minor length was also assessed. All assessments were conducted before the first training session of the day, under resting conditions, to minimize the effects of transient fatigue. During evaluation, participants wore swimwear to allow clear visualization of the scapula [19], and each shoulder was assessed independently [14].

### 2.3. Scapular Dyskinesis Test

**Figure 1:**
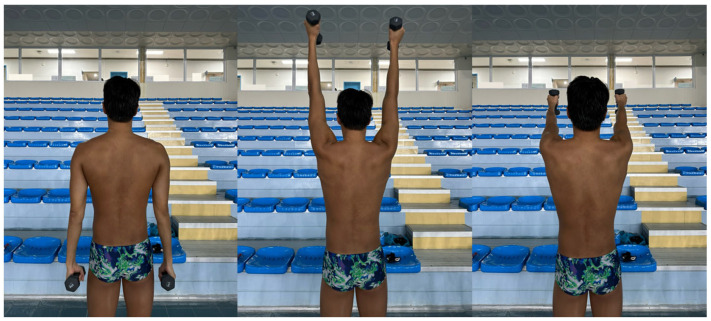
Procedure of the scapular dyskinesis test.

Participants weighing <68.1 and ≥68.1 kg held 1.5 kg or 2 kg dumbbells, respectively, in each hand. Standing with their thumbs pointing upward, participants elevated their shoulders to 180° over 3 s and then returned to 0° over 3 s; this movement was repeated five times (Figure 1). The examiner observed and recorded scapular motion from a distance of 2 m. Movements were classified as “normal,” “subtle,” and “obvious” if no, mild, and clear abnormalities, respectively, were present [20]. The reliability of SDT is almost perfect (k = 0.86) [21].

### 2.4. Lateral Scapular Slide Test

**Figure 2:**
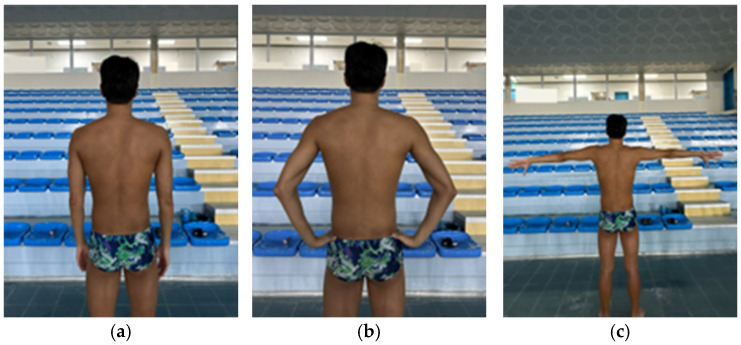
Lateral scapular slide test position. (**a**) arms at side; (**b**) hands on pelvis; (**c**) arms abducted to 90° with full internal rotation.

The examiner measured the distance between the spinous process of the seventh thoracic vertebra and the inferior angle of the scapula in three positions using a caliper (CAS, Yangju, Republic of Korea) [22]. The three positions were as follows: (1) arms relaxed at the sides, (2) hands placed on the pelvis with the thumbs pointing posteriorly and the remaining fingers pointing anteriorly, and (3) arms abducted to 90° with full IR (Figure 2) [23]. Each position was measured twice, and the mean value was used for analysis [24]. The reliability of LSST is reported excellent level (ICC > 0.92) [25].

### 2.5. Shoulder Internal and External Rotation Range of Motion

**Figure 3:**
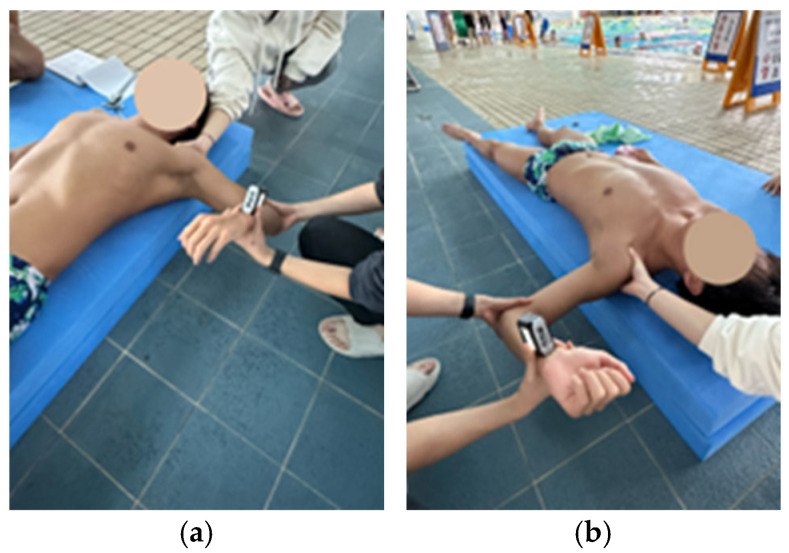
Measuring shoulder rotation range of motion. (**a**) internal rotation; (**b**) external rotation.

Internal and external rotation ROM were measured with participants in a supine position, the shoulder abducted to 90°, and the elbow flexed to 90° [26]. A digital inclinometer (EX-POWER, Ansan, Republic of Korea) was placed at the midpoint of the distal end of the forearm [26]. The first examiner stabilized the scapula by holding the coracoid process and spine of the scapula [27], while the second examiner rotated the humerus at the glenohumeral joint to achieve maximal passive rotation (Figure 3) [26]. The endpoint of the ROM was defined as the point at which scapular movement was detected [28]. All measurements were performed twice, and the mean value was used for analysis [28]. The reliability is reported excellent level (ICC = 0.96–0.99) [29].

### 2.6. Pectoralis Minor Length

**Figure 4:**
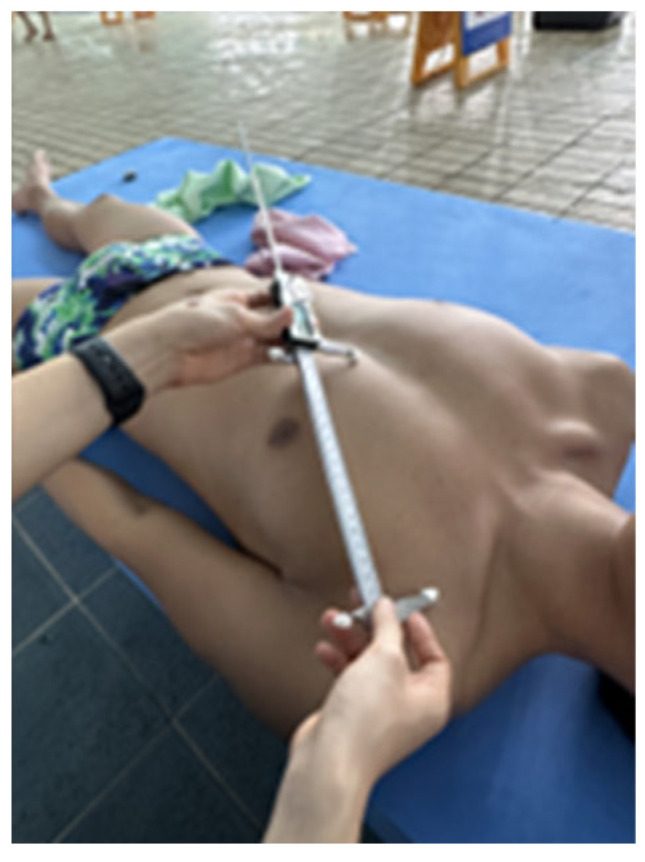
Measuring pectoralis minor length.

A caliper (CAS, Yangju, Republic of Korea) was used to measure the length of the pectoralis minor. The measurement was performed in the supine position by marking the medial-inferior angle of the coracoid process and the lateral sternal junction of the fourth rib, and then measuring the distance between these two points (Figure 4) [30,31]. Each measurement was performed twice, and the mean value was used for analysis [32]. The measured length was then standardized by dividing it by the participant’s height and multiplying by 100 [33]. All statistical analyses and data presentations were performed using standardized values. The reliability is good (ICC = 0.82–0.87) [30].

The examiner for SDT and all examiners for all measurements were blinded to each other. The reliability of the examiners was confirmed as substantial to almost perfect, including for the SDT (k = 0.78–0.81), LSST (ICC = 0.85–0.93), shoulder rotation ROM (ICC = 0.91–0.94), and pectoralis minor length measurement (ICC = 0.84).

### 2.7. Penn Shoulder Score

The shoulder pain, satisfaction, and functional level were assessed using the Penn Shoulder Score questionnaire, which is a 100-point scale comprising three subscales: pain, satisfaction, and function. Pain was evaluated for three conditions—at rest, during activities of daily living, and during strenuous activity—on a scale from 0 (no pain) to 10 (worst pain). For each item, the score was subtracted from 10, and the resulting values were summed to yield a pain subscale score ranging from 0 to 30, with 30 indicating no pain. Satisfaction was assessed as a single item on a scale from 0 (not satisfied) to 10 (very satisfied). Function was evaluated using 20 items, with each scored on a Likert scale from 0 to 3 points, with a maximum score of 60 indicating the ability to perform all activities without difficulty. A total of 100 points reflects low pain, high satisfaction, and excellent function. This questionnaire has demonstrated excellent reliability (ICC = 0.94) [34].

### 2.8. Statistical Analysis

All statistical analyses were performed using IBM SPSS Statistics version 29.0 (SPSS Inc., Chicago, IL, USA). Participant characteristics and swimming-related information were presented as descriptive statistics. The normality was assessed using the Shapiro–Wilk test. The relationships between years of experience and SDT score, and between SDT score and Penn Shoulder Score, were analyzed using Spearman’s correlation. Differences in SDT scores according to primary stroke were examined using the Kruskal–Wallis test whereas differences in LSST distance, shoulder IR-ROM, and pectoralis minor length according to SD severity were evaluated using one-way analysis of variance (ANOVA). When significant differences were found, Bonferroni post hoc tests were conducted. Associations between the presence or severity of SD and the presence of shoulder pain were analyzed using the chi-square test, and differences in Penn Shoulder Score according to the presence of SD were analyzed using the Mann–Whitney U test. The level of significance was set at *p* < 0.05. Effect sizes were reported along with statistical significance. According to Cohen’s criteria [35], partial eta squared (η^2^_p_) values of 0.01, 0.06, and 0.14 in one-way ANOVA were interpreted as small, medium, and large effects, respectively. For the Mann–Whitney U test and Spearman’s correlation, *r* values of 0.1, 0.3, and 0.5 were considered small, medium, and large effects, respectively.

## 3. Results

### 3.1. Anthropometric Data and Swimming Characteristics

A total of 50 adult swimmers (38 men and 12 women) participated in the study. The mean age was 23.96 ± 3.14 years, and the mean duration of swimming experience was 13.60 ± 3.74 years. Participants’ anthropometric data and swimming-related characteristics are summarized (Table 1 and Table 2).

### 3.2. The Prevalence of SD

Among the 50 participants, SD was observed in at least one shoulder in 23 swimmers (46.0%), with 9.0% obvious, 26.0% subtle, and 65.0% normal SD grades (Table 3). The prevalence of obvious SD was high on the dominant side (12.0%) compared with the non-dominant side (6.0%).

### 3.3. Association with Dominant and Breathing Side

**Figure 5:**
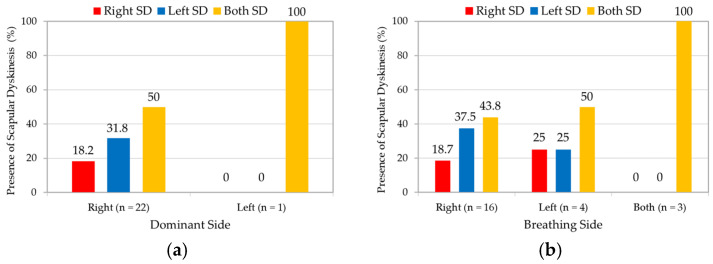
Directional distribution of scapular dyskinesis based on (**a**) dominant side and (**b**) breathing side. Both analyses were performed on 23 participants with scapular dyskinesis. SD, scapular dyskinesis.

In right-dominant swimmers, 50% (*n* = 11) showed bilateral SD, 18.2% (*n* = 4) showed only right shoulder SD, and 31.8% (*n* = 7) showed only left shoulder SD (Figure 5a). There was one left handed swimmer that showed bilateral SD. According to the breathing side, right-breathing swimmers showed 18.7% (*n* = 3) SD in only right shoulder, 37.5% (*n* = 6) in only left shoulder, and 43.8% (*n* = 7) showed bilateral SD (Figure 5b). Left-breathing swimmers showed 25% (*n* = 1) SD in only right shoulder, 25% (*n* = 1) in only left shoulder, and 50% (*n* = 2) showed bilateral SD. All three bilateral breathing swimmers showed bilateral SD.

### 3.4. Association with Years of Experience

Spearman’s correlation analysis revealed no significant association between years of experience and the SDT score (*r* = 0.127, *p* = 0.378) (Table 4). The SDT score was calculated as the mean value of both arms.

### 3.5. Comparison by Main Stroke Type

After excluding six swimmers who competed in multiple strokes, 44 swimmers were categorized by primary stroke. Obvious SD was observed only in the butterfly and freestyle groups. The mean rank of SDT scores (normal = 1, subtle = 2, obvious = 3) was highest in the butterfly group (32.92), followed by freestyle, individual medley, backstroke, and breaststroke. The Kruskal–Wallis test revealed no statistically significant differences among groups; however, a trend was observed (H = 8.323, *p* = 0.080) (Table 5).

### 3.6. Comparison of LSST Length, Pectoralis Minor Length, and IR-ROM

One-way ANOVA revealed no significant differences in LSST distances 1, 2, and 3 according to SD grade (F = 0.104, *p* = 0.901; F = 0.692, *p* = 0.503; F = 0.004, *p* = 0.996) (Table 6). In contrast, pectoralis minor length differed significantly across SD grade levels (F = 13.283, *p* < 0.001, η^2^_p_ = 0.215), representing a large effect size. The obvious group (8.98 ± 0.48) had significantly shorter lengths than both the normal group (9.96 ± 0.58) and the subtle group (9.65 ± 0.56; *p* < 0.001, d = 2.04; *p* = 0.008, d = 1.40), with large effect sizes. There was a tendency (*p* = 0.51) for the subtle SD group to show shorter pectoralis minor length than the normal group. There was a significant difference in IR-ROM with SD grade (F = 4.561, *p* = 0.013, η^2^_p_ = 0.090), which represented a medium effect size. The subtle group (66.00 ± 9.32) had significantly greater values than the normal group (60.60 ± 9.16) (*p* = 0.047, d = 0.58), which indicated a medium effect size, whereas the obvious group (56.19 ± 8.29) had significantly smaller values than the subtle group (*p* = 0.029, d = 1.18), which indicated a large effect size.

### 3.7. Comparison of Shoulder Pain

The chi-square test revealed no significant differences in the prevalence of shoulder pain according to the presence or severity of SD (χ^2^ = 1.193, *p* = 0.275; χ^2^ = 1.437, *p* = 0.488) (Table 7).

### 3.8. Comparison of Penn Shoulder Score

The Mann–Whitney U test showed that the mean rank of the Penn Shoulder Score was significantly higher in the normal group (29.41) than in the SD group (20.91; Z = −2.055, *p* = 0.039, *r* = 0.29), suggesting a small effect size that approached a medium effect (Table 8).

### 3.9. Association Between Penn Shoulder Score and SDT Score

Spearman’s correlation analysis showed that the Penn Shoulder Score was significantly and negatively correlated with both the dominant-side and bilateral SDT scores (*r* = −0.291, *p* = 0.040; *r* = −0.295, *p* = 0.037), which were interpreted as small effects that approached a medium effect (Table 9).

## 4. Discussion

SD was observed in 46% (*N* = 50) of the adult swimmers with a mean age of 23.9 years, which was similar to the 44% in collegiate swimmers with a mean age of 19.6 years [14]. In contrast, the prevalence of SD was 8.5% in adolescent swimmers with a mean age of 15.8 years [13] and 69% in swimmers with a mean age of 10.3 years [5]. These findings indicate that the prevalence of SD is not consistent across age groups.

Swimming involves a symmetrical use of both shoulders. Among swimmers with a right-dominant side, SD was most frequently observed bilaterally, and least on the dominant side. This finding aligns with previous research reporting no association between the direction of dominance and the side of SD occurrence; however, this symmetry may not be maintained in swimmers favoring a specific breathing side [13]. Consistent with the trends in previous studies [13], among right-breathing swimmers, SD occurred more frequently on the contralateral side than on the breathing side; all swimmers with bilateral breathing exhibited bilateral SD. The breathing-side shoulder spends 14% more time in the pull phase, 16% less time in the push phase, and achieves 6% less hand depth [36]. Thus, the contralateral shoulder is potentially subjected to continuous functional stress throughout the push phase; repeated IR of the scapula on the non-breathing side during breathing may contribute to functional SD [13]. Owing to the limited number of swimmers with SD, no statistical tests were performed; further studies with larger cohorts are needed to confirm these findings.

No significant association was found between years of experience and SDT score, in contrast with a study of swimmers with >4 years of experience and twice the likelihood of SD (mean age 15.8 years; mean career length 6.4 years) [13]. Swimming demands early specialization and pre-pubertal high-intensity training [37], and repetitive upper-extremity overload during swim training induce SD in the short term [16,19] and, over many years, may contribute to the development of abnormal scapular movement patterns. In swimmers who began training early and have accumulated long careers, additional years of experience potentially confers a limited effect on SD occurrence. Future studies to longitudinally track SD prevalence by years of experience in the same cohort are needed.

No statistically significant differences in SDT scores were found by primary stroke; however, a consistent trend was observed (*p* = 0.08). The butterfly stroke had the highest mean rank of SDT scores, and obvious SD was observed only in the butterfly and freestyle groups. During the recovery phase, shoulder flexion and IR, and anterior translation of the humeral head owing to arm hyperextension at the end of the pull-through phase, may contribute to the impingement syndrome [38]. In butterfly and freestyle, these repetitive shoulder movements can exacerbate impingement involving the supraspinatus and the long head of the biceps tendon [39]. Individuals with impingement exhibit decreased scapular upward rotation, increased anterior tilt, and increased IR during arm elevation [40]. These alterations in scapular kinematics are key factors in the onset of symptoms of impingement syndrome [41], and may contribute to greater SD grade. This relationship has clinical relevance in that swimmers performing butterfly and freestyle strokes are prone to scapular dyskinesis associated with repetitive impingement mechanisms. Freestyle accounts for a large proportion of training regardless of the primary stroke [1,42], which partly explains the absence of significant differences in SD among strokes. The unequal sample sizes across stroke types and the limitations of non-parametric testing possibly limited the ability to fully capture the distribution of SDT scores, which warrants further investigation.

No significant differences were observed in LSST distances by SD grade. The LSST 2 distance in the normal group was significantly shorter than in the subtle and obvious groups [43]. Measured in a static posture, the LSST may not fully reflect scapular kinematics during functional movements [24]. Significant positive correlations between the LSST distance and trapezius, serratus anterior strength have been reported [44], whereby muscle strength may influence LSST measurements. Thus, scapular function evaluation in athletes should include dynamic and static indicators [24]. Besides LSST findings, pectoralis minor length significantly decreased with increasing SD severity, as in a study of boxers [45]; other research reported shorter pectoralis minor length in participants with SD [32]. Pectoralis minor shortening restricts scapular upward rotation, external rotation, and posterior tilt, and thereby impairs normal scapular motion [33]. No significant differences in LSST distances were observed across severity groups, despite the theoretical link with pectoralis minor shortening. A study applying muscle-energy techniques to swimmers found that pectoralis minor length increased without changes in scapular upward rotation [46]; thus, the direct relationship between pectoralis minor length and LSST distance may not be pronounced in athletes. Future studies with a wider range of shoulder kinematic variables are needed to more precisely determine this relationship.

The obvious group demonstrated significant IR-ROM reduction than the subtle group and non-significant decrease relative to the normal group. This finding differs from previous research reporting that decreased IR-ROM during arm movement induces “scapular wind-up” through tension in the posterior capsular structures, leading to SD and related issues [47]. The significant IR-ROM reduction in the obvious group is potentially associated with increasing SD severity; however, a linear decreasing trend across severity levels was not identified. This nonlinear pattern was potentially influenced by the presence or absence of symptoms. Shoulder kinematics of asymptomatic patients with rotator-cuff tears resembled those of healthy shoulders, and not of symptomatic patients [48]. Thus, functional capacity can be maintained despite structural abnormalities, and our subtle group possibly represents such a condition. Future research should include analyses stratified by symptom status to clarify the relationship between IR-ROM and SD severity.

No significant differences were found in the prevalence of shoulder pain according to the presence or severity of SD. This finding is consistent with previous research on adolescent athletes, which reported no association between SD and shoulder pain [49]. Studies of overhead athletes have indicated that scapular anterior tilt and winging are not risk factors for shoulder pain [50]. In contrast, other reports have suggested that a scapular position closer to the spine increases the risk of shoulder pain [51] and that the presence of SD in asymptomatic athletes increases the risk of developing shoulder pain by 43% [52]. Such discrepancies may be attributable to differences in sport type, participant age, and methods of SD assessment. Swimmers with SD demonstrated significantly lower Penn Shoulder Score values, and SDT scores showed significant negative correlations with the Penn Shoulder Score on both the dominant and bilateral sides. Thus, SD is potentially associated with subjective shoulder pain, discomfort, and functional decline. No association was found between SD and the prevalence of shoulder pain; however, the association with the Penn Shoulder Score suggests that SD may represent not solely a pain-driven pathology, but potentially a normal functional adaptation or change from repetitive stroke activity [53]. Although SD observed in this study may represent an adaptive or compensatory response, its underlying biomechanical mechanisms overlap with those implicated in shoulder pathologies such as GIRD, and SICK scapula syndrome [8,9]. Therefore, identifying functional scapular alterations in swimmers is clinically relevant for the early detection or prevention of secondary shoulder disorders that may develop from persistent scapular dysfunction.

This study has some limitations. First, owing to its observational cross-sectional design, causal relationships between SD and anatomical or functional characteristics, including shoulder pain, could not be established; longitudinal follow-up studies are needed to confirm such associations. Second, the division of participants into three groups according to SD severity created unequal sample sizes; the small sample in the obvious SD group potentially reduced statistical power. Third, despite a highly reliable method for SD assessment, the classification process relied on the examiner’s experience and judgment. Fourth, training variables, such as volume, intensity, and frequency, were not considered, and their influence on SD occurrence remains undetermined. Fifth, as the study included only adult elite swimmers, the findings may have limited generalizability to other age groups. Finally, although the LSST is sensitive for identifying grade II SD, its ability to detect grade I cases is limited and should be acknowledged as a measurement limitation.

## 5. Conclusions

In conclusion, SD was observed in 46% of adult elite swimmers. Years of experience and primary stroke were not significantly associated with the occurrence of SD; however, obvious SD appeared only in butterfly and freestyle. Greater SD severity was linked to reduced pectoralis minor length and decreased IR-ROM, particularly in the obvious group, suggesting potential functional limitations with severe SD grade. SD was unassociated with pain, but significantly related to subjective shoulder function.

## Figures and Tables

**Table 1:** Anthropometric data.

	Male (*n* = 38)	Female (*n* = 12)	Total (*N* = 50)
Age (y)	23.39 ± 2.55	25.75 ± 4.16	23.96 ± 3.14
Height (cm)	179.39 ± 5.58	165.67 ± 4.66	176.10 ± 7.96
Weight (kg)	75.18 ± 5.01	58.50 ± 5.68	71.18 ± 8.83
Hand dominance (*n*, %)			
Right	35 (92.1)	12 (100.0)	47 (94.0)
Left	3 (7.9)	0 (0.0)	3 (6.0)

Values are presented as mean ± standard deviation or as *n* (%).

**Table 2:** Swimming characteristics.

	Male (*n* = 38)	Female (*n* = 12)	Total (*N* = 50)
Years of experience (y)	13.26 ± 3.17	14.67 ± 5.20	13.60 ± 3.74
Breathing side (*n*, %)			
Right	30 (78.9)	8 (66.7)	38 (76.0)
Left	3 (7.9)	1 (8.3)	4 (8.0)
Both	5 (13.2)	3 (25.0)	8 (16.0)
Stroke (*n*, %)			
Butterfly	8 (19.1)	3 (21.4)	11 (19.6)
Backstroke	8 (19.1)	0 (0.0)	8 (14.3)
Breaststroke	8 (19.1)	1 (7.2)	9 (16.1)
Freestyle	15 (35.6)	7 (50.0)	22 (39.3)
Individual Medley	3 (7.1)	3 (21.4)	6 (10.7)
Distance (*n*, %)			
Sprint	26 (46.4)	4 (18.2)	30 (38.4)
Mid-distance	24 (42.9)	12 (54.5)	36 (46.2)
Long-distance	6 (10.7)	6 (27.3)	12 (15.4)

Values are presented as mean ± standard deviation or as *n* (%). Noth that the number of participants (*N*) may exceed 50, as some swimmers specialized in more than two strokes or distances.

**Table 3:** The prevalence of SD in swimmers.

	Normal	Subtle	Obvious
Dominant arm (*n* = 50)	34 (68.0%)	10 (20.0%)	6 (12.0%)
Non-dominant arm (*n* = 50)	31 (62.0%)	16 (32.0%)	3 (6.0%)
Total (*N* = 100)	65 (65.0%)	26 (26.0%)	9 (9.0%)

Values are presented as *n* (%). SD, scapular dyskinesis.

**Table 4:** Correlation between years of experience and the SDT score.

	*r*	*p*
Years of experience and SDT score	0.127	0.378

SDT, scapular dyskinesis test.

**Table 5:** Severity distribution and mean ranks of SDT score by main stroke type.

	Normal	Subtle	Obvious	Mean Rank	Kruskal–Wallis H	*p*
Butterfly(*n* = 12)	4 (33.3%)	4 (33.3%)	4 (33.3%)	32.92	8.323	0.080
Backstroke (*n* = 14)	12 (85.7%)	2 (14.3%)	0 (0.0%)	17.86		
Breaststroke (*n* = 18)	15 (83.3%)	3 (16.7%)	0 (0.0%)	17.50		
Freestyle (*n* = 38)	23 (60.5%)	11 (29.0%)	4 (10.5%)	23.89		
Individual Medley (*n* = 6)	5 (83.3%)	1 (16.7%)	0 (0.0%)	18.67		

Values are presented as *n* (%). SDT, scapular dyskinesis test.

**Table 6:** Comparison of LSST length, pectoralis minor length, IR-ROM by SD severity.

	Normal ^a^ (*n* = 65)	Subtle ^b^ (*n* = 26)	Obvious ^c^ (*n* = 9)	F	*p*	Effect Size (η^2^_p_)	Bonferroni
LSST 1 (cm)	11.32 ± 0.94 (11.09–11.55)	11.21 ± 1.28 (10.69–11.73)	11.27 ± 1.04 (10.47–12.06)	0.104	0.901		
LSST 2 (cm)	12.33 ± 0.99 (12.08–12.57)	12.02 ± 1.42 (11.45–12.59)	12.21 ± 1.17 (11.31–13.11)	0.692	0.503		
LSST 3 (cm)	12.58 ± 1.04 (12.32–12.84)	12.60 ± 1.11 (12.15–13.04)	12.56 ± 1.42 (11.47–13.65)	0.004	0.996		
Pec m length	9.96 ± 0.58 (9.82–10.11)	9.65 ± 0.56 (9.42–9.87)	8.98 ± 0.48 (8.61–9.35)	13.283	<0.001 ***	0.215	a > b (0.051) b > c (0.008 **) a > c (<0.001 ***)
IR ROM (°)	60.60 ± 9.16 (58.30–62.91)	66.00 ± 9.32 (62.06–69.94)	56.19 ± 8.29 (49.26–63.12)	4.561	0.013 *	0.090	a < b (0.047 *) b > c (0.029 *)

Values are presented as mean ± standard deviation (95% confidence interval). For the analysis of IR-ROM, three participants were excluded to secure normality, resulting in a sample size of 47. SDT, scapular dyskinesis test; LSST, lateral scapular slide test; Pec m, pectoralis minor; IR, internal rotation; ROM, range of motion. * *p* < 0.05, ** *p* < 0.01, *** *p* < 0.001.; a = normal, b = subtle, c = obvious.

**Table 7:** Comparison of shoulder pain according to the presence and severity of SD.

	**Normal**			**SD**			**χ^2^**	***p***
Shoulder pain (*n* = 33)	19 (29.2%)			14 (40.0%)			1.193	0.275
No Shoulder pain (*n* = 67)	46 (70.8%)			21 (60.0%)				
	**SDT Normal**		**SDT Subtle**		**SDT Obvious**		**χ^2^**	***p***
Shoulder pain (*n* = 33)	19 (29.2%)		11 (42.3%)		3 (33.3%)		1.437	0.488
No Shoulder pain (*n* = 67)	46 (70.8%)		15 (57.7%)		6 (66.7%)			

Values are presented as *n* (%). SD, scapular dyskinesis; SDT, scapular dyskinesis test.

**Table 8:** Comparison of Penn Shoulder Score according to the presence of SD.

	Mean Rank	Mann–Whitney U	*Z*	*p*
Normal (*n* = 27)	29.41	205	−2.055	0.039 *
SD (*n* = 23)	20.91			

SD, scapular dyskinesis. * *p* < 0.05.

**Table 9:** Correlation between the Penn Shoulder Score and SDT score.

	*r*	*p*
Dominant arm (*n* = 50)	−0.291	0.040 *
Non-dominant arm (*n* = 50)	−0.225	0.116
Both (*N* = 100)	−0.295	0.037 *

* *p* < 0.05.

## Data Availability

The original contributions presented in this study are included in the article. Further inquiries can be directed to the corresponding author.

## Abbreviations

The following abbreviations are used in this manuscript:

SD	Scapular dyskinesis
LSST	Lateral scapular slide test
IR	Internal rotation
ROM	Range of motion
SDT	Scapular dyskinesis test
